# History of Cobaltabis(dicarbollide) in Potentiometry, No Need for Ionophores to Get an Excellent Selectivity

**DOI:** 10.3390/molecules27238312

**Published:** 2022-11-29

**Authors:** Anca-Iulia Stoica, Clara Viñas, Francesc Teixidor

**Affiliations:** 1 Department of Water-Atmosphere Resources and Life Science, University of Natural Resources and Life Sciences, 1180 Vienna, Austria; 2 Institut de Ciencia de Materials de Barcelona, ICMAB-CSIC, 08193 Bellaterra, Spain

**Keywords:** Ion-Selective Electrodes, potentiometry, ion pair complexes, cobaltabis(dicarbollide), nitrogen containing compounds

## Abstract

This work is a mini-review highlighting the relevance of the θ metallabis(dicarbollide) [3,3′-Co(1,2-C_2_B_9_H_11_)_2_]^−^ with its peculiar and differentiating characteristics, among them the capacity to generate hydrogen and dihydrogen bonds, to generate micelles and vesicles, to be able to be dissolved in water or benzene, to have a wide range of redox reversible couples and many more, and to use these properties, in this case, for producing potentiometric membrane sensors to monitor amine-containing drugs or other nitrogen-containing molecules. Sensors have been produced with this monoanionic cluster [3,3′-Co(1,2-C_2_B_9_H_11_)_2_]^−^. Other monoanionic boron clusters are also discussed, but they are much fewer. It is noteworthy that most of the electrochemical sensor species incorporate an ammonium cation and that this cation is the species to be detected. Alternatively, the detection of the borate anion itself has also been studied, but with significantly fewer examples. The functions of the borate anion in the membrane are different, even as a doping agent for polypyrrole which was the conductive ground on which the PVC membrane was deposited. Apart from these cases related to *closo* borates, the bulk of the work has been devoted to sensors in which the θ metallabis (dicarbollide) [3,3′-Co(1,2-C_2_B_9_H_11_)_2_]^−^ is the key element. The metallabis (dicarbollide) anion, [3,3′-Co(1,2-C_2_B_9_H_11_)_2_]^−^, has many applications; one of these is as new material used to prepare an ion-pair complex with bioactive protonable nitrogen containing compounds, [YH]_x_[3,3′-Co(1,2-C_2_B_9_H_11_)_2_]_y_ as an active part of PVC membrane potentiometric sensors. The developed electrodes have Nernstian responses for target analytes, i.e., antibiotics, amino acids, neurotransmitters, analgesics, for some decades of concentrations, with a short response time, around 5 s, a good stability of membrane over 45 days, and an optimal selectivity, even for optical isomers, to be used also for real sample analysis and environmental, clinical, pharmaceutical and food analysis.

## 1. Introduction, Objectives and Characteristics of *Closo*-Borates and Metallabis(dicarbollides)

This mini-review deals with the potentiometric application of metallabis(dicarbollides) in detecting basic nitrogen containing compounds, mostly in some pharmaceuticals. Thus it does not provide a summary of the organic chemistry of nitrogenous compounds, which is very extensive; according to Jonnalagadda et al. [1], there have been over 97,400 papers only dedicated to nitrogen heterocycles between 2009 and early 2020, nor on the wide list of top prescribed drugs containing nitrogen heterocycles that has been comprehensively reviewed [2], although a large proportion of these could be target compounds to be analyzed by the potentiometric method reported here; it does not deal on analytical techniques on drug analysis, that have been well reviewed [3], or more specifically on the application of electrochemical methods for pharmaceutical or drug analysis [4]. Concerning potentiometric sensors, more specifically Ion Selective Electrodes (ISEs), have been broadly employed as one of the most important electrochemical approaches for pharmaceutical drug analysis [5,6]. Since the advent of nanoscience, nanomaterial components and concepts are available that can improve the design of ISEs [7,8,9], thus it is expected that a new momentum for the fabrication of selective ISEs and nanomaterials-based potentiometric platforms for pharmaceutical drug analysis will take place. In this work, we will prove that by using metallabis(dicarbollides) it is possible to design and manufacture very selective, very stable, long-lasting, ionophore-free ISEs, without internal solutions and reference electrodes in the working electrode; all thanks to the unique characteristics of metallabis(dicarbollides).

Some years ago, little was known about the properties in a solution of the metallabis(dicarbollide) cobaltabis(dicarbollide) anion [3,3′-Co(1,2-C_2_B_9_H_11_)_2_]^−^ [10], also known as [*o*-COSAN]^−^, which possess the two C atoms connected. Most efforts had been devoted to its synthesis and derivatization, although its redox reversibility was well known. There were three known redox couples, Co^4+/3+^, Co^3+/2+^, and Co^2+/1+^, each of them reversible [11,12,13]. 

**Figure 1:**
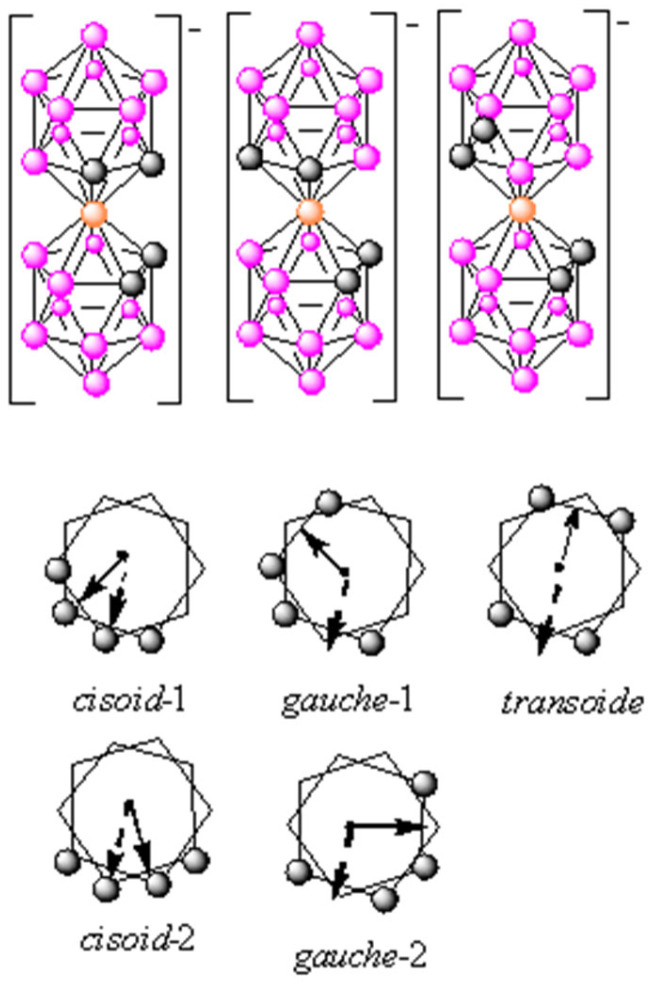
Schematic representation of the icosahedral anionic cobaltabis(dicarbollide) cluster [3,3′-Co(1,2-C_2_B_9_H_11_)_2_]^−^ conformers (the arrows indicate the direction of dipole moments of compounds). Circles in grey represent the C_c_-H vertices; the orange ones correspond to metal (M = Co^3+^, Fe^3+^), while the circles in pink correspond to B-H vertices.

The molecule has a structure that, while not rigid, does not change volume or shape. Its shape resembles the theta letter, θ [14], which allows a non-free rotation around the cobalt atom, so that three types of conformers can be generated, *transoid*, *cisoid*, and *gauche* [15]. As recently demonstrated, the three conformers show different properties depending on the environment. If the environment is polar, water, or in the presence of ions, the *cisoid* conformer is prevalent. If it is in vacuum or non-polar solvents, the *transoid* conformer is dominant [16]. Therefore, depending on the environment, the properties in the solution are very different. Thus, in aqueous media, the space around the 4 C_cluster_-Hs is hydrophobic while the space around the 18 B-Hs is hydrophilic [15]. Apart from the hydrophobic/hydrophilic interactions, non-covalent hydrogen and dihydrogen interactions play a key role [17]. Figure 1 displays the five conformers of the isomer [3,3′-Co(1,2-C_2_B_9_H_11_)_2_]^−^, abbreviated as [*o*-COSAN]^−^, which are *cisoid*-1, *gauche*-1, *transoid*, *gauche*-2, and *cisoid*-2 (being *cisoid*-1 and *cisoid*-2 as well as *gauche*-1 and *gauche*-2 equivalent.

These interactions explain why aggregates, vesicles, or micelles with monomers coexist in an aqueous solution [14]. This amphiphilic behavior also explains that [*o*-COSAN]^−^ can pass through cell membranes [18]. Apart from this type of interaction, the θ cobaltabis(dicarbollide) [*o*-COSAN]^−^ interacts strongly with proteins as demonstrated by the interaction with Bovine Serum Albumin BSA [19]. In this case, there are about one hundred [3,3′-Co(1,2-C_2_B_9_H_11_)_2_]^−^ units per each BSA protein. This amount of cobaltabis(dicarbollide) per BSA coated the entire surface of the BSA that was interpreted by considering two phenomena: the anchoring capacity of [*o*-COSAN]^−^ with amino acids whose residue contained amino groups, i.e., Lysine, Arginine, and Histidine, and the self-assembly capacity of the [*o*-COSAN]^−^ anions. The cobaltabis(dicarbollide) anion can dissolve in very non-polar and very polar media depending on the cation, but in the case of H [3,3′-Co(1,2-C_2_B_9_H_11_)_2_], it is soluble from benzene to water. This makes it a unique compound. 

## 2. Generalities of Ion Selective Electrodes (ISEs) and First Steps in the Use of *Closo* Borates and Metallabis(dicarbollides) as ISEs

Ion Selective Electrodes (ISEs) are transducers or sensors that convert the activity of a specific ion dissolved in a solution into an electrical potential, which can be measured by a voltmeter. ISEs have different applications in clinical, pharmaceutical, environmental, and food processing industries [6,20,21,22,23,24] due to their efficiency from an economical point of view, and analysis time. These sensors are related to low price, and following work due to Bloch, Simon, and Thomas on PVC=based membranes, their performance was improved a lot concerning the limit of detection and selectivity [25,26,27].

In 1999, [3,3′-M(1,2-C_2_B_9_H_11_)_2_]^−^ (M = Co^3+^, Fe^3+^, Ni^3+^) compounds were implemented in PVC membranes to study their performance as Cs^+^ sensors in ISEs. The three metallabis(dicarbollide) complexes displayed a similar behavior with a near-Nernstian response close to 51 mV decade^−1^ and, the [3,3′-Co(1,2-C_2_B_9_H_11_)_2_]^−^ anion was chosen as the parent on which C-substitution, both alkyl and aromatic were done. The species [3,3′-Co(1-CH_3_-2-(CH_2_)_n_ OR-1,2-C_2_B_9_H_9_)_2_]^−^ ([1]^−^: n = 3, R = –CH_2_CH_3_; [2]^−^: n = 3, R = –(CH_2_)_2_OCH_3_; [3]^−^: n = 3, R = −(CH_2_)_3_CH_3_; [4]^−^: n = 6, R= −(CH_2_)_3_CH_3_), and [3,3′-Co(1-C_6_H_5_-1,2-C_2_B_9_H_10_)_2_]^−^ ([5]^−^) and [3,3′-Co(1,7-(C_6_H_5_)_2_-1,7-C_2_B_9_H_9_)_2_]^−^ ([6]^−^) were tested for ^137^Cs, ^90^Sr, and ^152^Eu in extraction as long as for potentiometric detection of Cs. In addition, permeability tests on supported liquid membranes with H [6], H [4], and H [6] showed that these compounds present the highest values reported so far for this sort of radionuclide transport experiment [28].

By the year 2006, when we initiated our potentiometric work on using metallabis(dicarbollides) to generate electroactive salts for the selective determination of amine-containing relevant drugs, two key experimental data were known for synthetic boron cluster chemists that were very relevant to start this research: the water insolubility of salts of Cs, used for Cs^+^ sensing [28] and alkylammonium with anionic boron clusters and that these same salts were soluble in organic solvents like THF. This concept has been utilized with another series of *closo* boranes, e.g., tetradecylammonium triethylammonium-*closo*-dodecaborate as the electroactive species to determine [B_12_H_11_N(C_2_H_5_)_3_]^−^ [29], or the use of the sulfonium derivative of the *closo*-hydridodecaborate anion [B_10_H_9_S(C_18_H_37_)_2_]^−^ as the active component for a potentiometric lidocaine-selective sensor [30]. Concerning the use of the *closo* borate clusters the 2, 3, 4, 5, 6, 7, 8, 9, 10, 11, 12-undecabromocarborane anion, [1-HCB_11_Br_11_]^−^ has been studied as an alternative to the best lipophilic tetraphenylborate, 3,5-[bis(trifluoromethyl)phenyl]borate demonstrating a much higher persistence in the potentiometric membrane [31]. 

**Figure 2:**
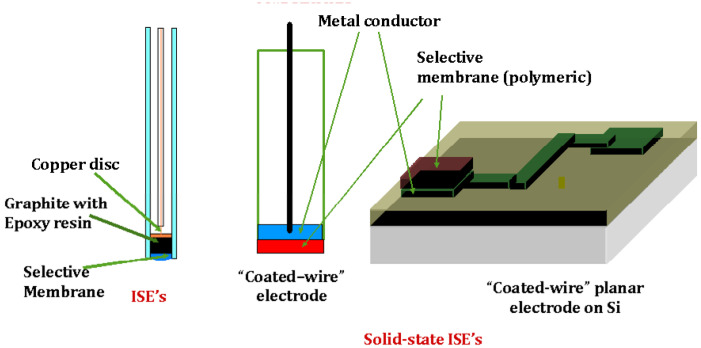
Schematic representation of solid ISEs (**left**) and the similar coated-wire or Si wafer (**right**).

Common key components of a membrane ISE are an inner reference solution on one side of the membrane, a second reference electrode in contact with the analyte solution, and the membrane itself on which at each interface is established an ion-exchange equilibrium that results in charge separation at each interface producing a phase-boundary potential [32,33]. When concentrations of the ion to be measured on both sides of the membrane are not equal, a membrane potential develops. The difference in potential is measured by the two reference electrodes. There are four major types of membrane: glass, crystalline, liquid, and polymer. The last two are also known as ion exchange membranes. In this mini-review, we mostly dedicate to the polymer type in which the selective membrane consists of three main components: ionophores, a polymer matrix, and a plasticizer. Figure 2 shows a schematic representation of the ISEs with PVC membrane in the solid-state, left, and on the right with a polypyrrol support [34] that performs as the conducting material on which stay the Ionophores, within the PVC membrane, which can be ionic or neutral; these are complexing agents capable of reversibly binding ions. Typically, solid membranes contain an ionophore, ion-exchanger additives (i.e., either alkylammonium salts for anion sensing or tetraphenylborates for cation sensing) [35,36], and a plasticizer which is the organic medium that is supposed to allow the transport of charges within the membranes. Instead of the ionophore, some membranes contain one ion pair complex, the anion part typically being tetraphenylborate and the cation part, the protonated analyte to be measured as the electroactive substance [37,38,39,40,41]. The ion pair complexes are charged ionophores, which bind ions in a more complex binding than mere electrostatic interaction, show increased selectivity towards a primary ion, and possess high strength of association constants [42,43].

**Figure 3:**
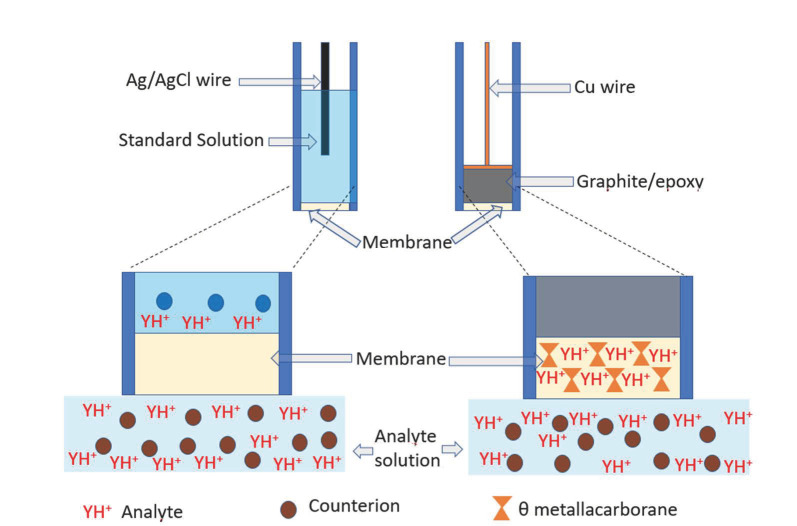
Schematic representation of conventional membrane liquid ISEs (**top left**) and membrane solid state (**top right**) with a magnification of their lower part displaying where the analytes are, and where the metallabis(dicarbollides) is.

Very relevant for this research on potentiometry based on *closo*-borate anions was that all PVC membranes for potentiometric sensors were prepared in THF. Therefore, the ammonium salts of [3,3′-Co(1,2-C_2_B_9_H_11_)_2_]^−^ and PVC had the same solubility requirements: very soluble in THF and insoluble in water. From the point of view of making the membrane, it seemed that all factors were pointing in the same direction. If now we restrict to the θ cobaltabis(dicarbollide) [3,3′-Co(1,2-C_2_B_9_H_11_)_2_]^−^ potentiometric electrodes and to make the construction simpler, even at the cost of having a lower limit of detection, LOD, we have moved away from the traditional electrode in which the membrane separated the solution with the analyte from an internal aqueous solution in which there was an inner reference electrode, see Figure 3 top. In 2006, the research addressed developing polymeric sensing membranes based on the principles of host-guest chemistry, as they allowed the selectivity of the sensor to be modulated. Many hundreds of receptors have been developed for this purpose. Typically, there was a lipophilic ion exchanger in addition to the ionophore, which was the gateway for the ions to be measured to enter the membrane. Commonly tetraphenylborate had been used as an ion exchanger, but also the boron cluster perbrominated *closo*-dodecacarborane anion, [1-HCB_11_Br_11_]^−^ had been used for this purpose [31]. It was then considered that the emf response of such membranes was described in a simple way by the phase boundary model [44,45,46], which assumes a localized equilibrium across interfaces and does not consider changes in potential within the membrane or the sample solution [47,48]. A view is shown in Figure 3, bottom. The applied equation is:

$$E_{PB}=E_{I}^{0}+\frac{RT}{z_{I}F}\ln\frac{a_{1}(aq)}{a_{1}(org)}$$

where *a_I_*(*aq*) and *a_I_*(*org*) are the activity of the ion *I* (with charge *z_I_*) in the aqueous and organic phase boundaries, and are derived from the chemical standard potentials in either phase. 

The above equation is reduced to the Nernst equation if the activity of the ion to be studied is constant in the organic phase. This required the presence of a lipophilic ion exchanger in the membrane [44,45,46] otherwise, the membrane would lose its selectivity [47,48].

$$emf=K+\frac{RT}{z_{I}F}\ln a_{I}(aq)$$

## 3. The Metallabis(dicarbollide) [3,3′-Co(1,2-C_2_B_9_H_11_)_2_]^−^ as an Active Component of Membrane Solid State ISE

Concerning the use of θ cobaltabis(dicarbollide) [3,3′-Co(1,2-C_2_B_9_H_11_)_2_]^−^ we assumed the basic concept of the rationale behind the recognition of ISE membranes, to suggest the build-up of a potential difference between the bulk of the membrane and the outer analyte aqueous phase (Figure 3 bottom right). The [3,3′-Co(1,2-C_2_B_9_H_11_)_2_]^−^ anion provides stability to all participating agents in the membrane. The novelty of this strategy is that the anion [3,3′-Co(1,2-C_2_B_9_H_11_)_2_]^−^ is not the sensing part, but the cation ([cation-NH]^+^) that leads to the selectivity. But this cation is strongly interacting with [3,3′-Co(1,2-C_2_B_9_H_11_)_2_]^−^, unlike tetraphenylborate to illustrate with a relevant example. For the latter, only electrostatic interactions are expected, but for [3,3′-Co(1,2-C_2_B_9_H_11_)_2_]^−^ in addition to these, hydrogen and dihydrogen bonds occur. Therefore, ion-pair complexes of this type do not fit with the traditional definition of ionophore; hence the importance of this unique cobaltabis(dicarbollide) anion in (bio)sensors.

**Figure 4:**
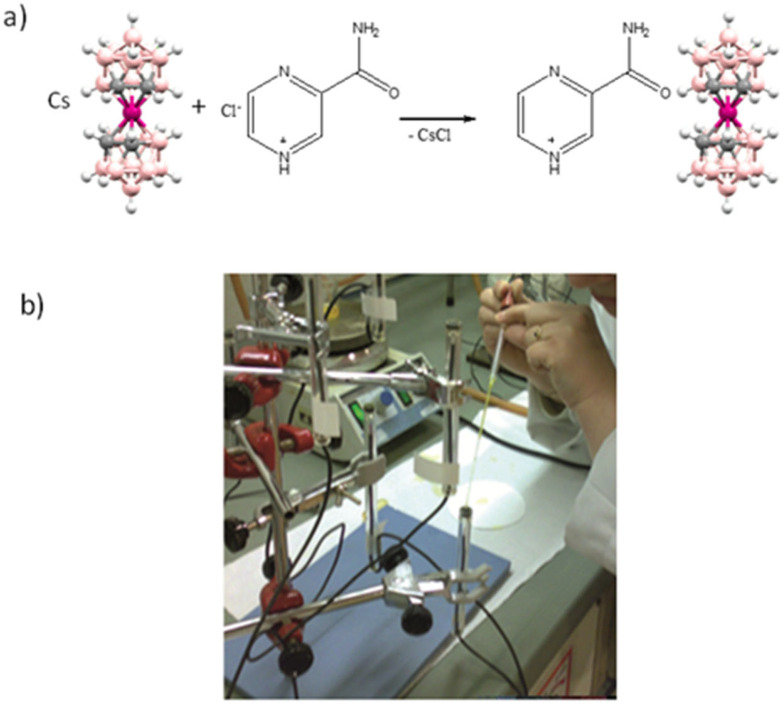
(**a**) Synthesis of the electro-active [YH]_x_[3,3Co(1,2-C_2_B_9_H_11_)_2_]_y_ salt for pyrazinamide (PZA). (**b**) Photo showing the simplicity of the ISE electrode preparation.

Therefore, we had an ion exchanger, [3,3′-Co(1,2-C_2_B_9_H_11_)_2_]^−^, the PVC, and the plasticizer, all within the membrane, and remarkably it was not necessary to design and synthesize complex molecules that would be selective for a given analyte Y. This would represent a major breakthrough as any protonable amine could be eligible as a candidate to be measured and certainly, it would represent a readily available source of electrochemical sensors while retaining selectivity as far as we were concerned. The presence of Y, in the membrane at the appropriate concentration, could already be achieved by adding the ion-pair complex [YH][3,3′-Co(1,2-C_2_B_9_H_11_)_2_], and there was confidence that YH^+^ would not leak out of the membrane. Most biologically active compounds have in their structure one or more amino groups that are able to be protonated, thus, our target analytes were compounds with pharmaceutical and medical applications, i.e., antibiotics, amino acids, neurotransmitters, analgesics, etc. [49,50,51,52,53,54,55,56]. Figure 4 displays the schematic general synthetic procedure of the electro-active [cation-NH]_x_[3,3′-Co(1,2-C_2_B_9_H_11_)_2_]_y_ salt, as well as the ISE electrode assembling.

It remained to be demonstrated whether this simplicity in the electrochemical sensor allowed for the detection of optically active species. Since the work of Simon et al. in 1975 [57], some papers and reviews have appeared dedicated to electrochemical enantioselective sensors and biosensors based on molecular chiral receptors, such as cyclodextrins, calixarenes, calixresorcinarenes, and crown-ethers, to form a complex preferentially with one of the enantiomers [58,59,60,61,62,63]. An important achievement of the [3,3′-Co(1,2-C_2_B_9_H_11_)_2_]^−^ in the membrane preparation for ISEs was the possibility to prepare ion pair complexes for enantiomers that were able to differentiate with a good selectivity of one enantiomer in the presence of the second one without using a chiral receptor, and this turned out to be possible [50]. This unexpected result after comparing with the current techniques described earlier to differentiate enantiomers must be a consequence of the strong interactions displayed by [3,3′-Co(1,2-C_2_B_9_H_11_)_2_]^−^ with the enantiomer in the membrane that prevents its fast rotation and mobility and therefore facilitates a better recognition. Recently chiral sensing systems based on chiral inorganic platforms have been reported for electrochemical recognition of enantiomers [64,65,66]. Also, [3,3′-Co(1,2-C_2_B_9_H_11_)_2_]^−^ has been used in the development of ISEs for the analysis of tropane alkaloids (tropane, atropine, and scopolamine [67], the analysis of antipyrine and its metabolites/derivatives from environmental water monitoring, which are (besides their beneficial health effect) of growing concern based on their occurrence and fate in water and the environment [56] as well as for serotonin detection [52].

**Figure 5:**
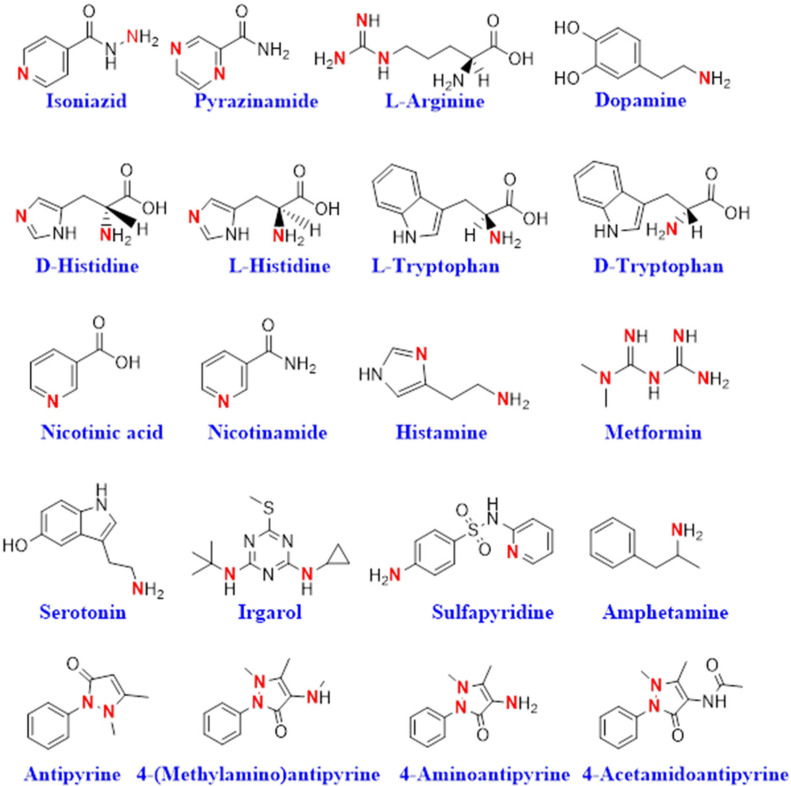
Chemical structures of bioactive compounds used to prepare [YH]_x_[3,3′-Co(1,2-C_2_B_9_H_11_)_2_]_y_. In red color were the amino groups that were expected to be basic enough to generate stable ion-pairs with [*o*-COSAN]^−^.

The chemical structures of bioactive nitrogen-containing compounds used in the ion-pair approach with [3,3′-Co(1,2-C_2_B_9_H_11_)_2_]^−^ had the formula [cation-NH]_x_[3,3′-Co(1,2-C_2_B_9_H_11_)_2_]_y_ and some of the reported amines analyzed to date are presented in Figure 5. In this figure, the amino groups that were expected to be basic enough to generate the stable ion pair with [3,3′-Co(1,2-C_2_B_9_H_11_)_2_]^−^ have been highlighted in red.

The x and y values in the formula of the ion-pair complex, [cation-NH]_x_[3,3′-Co(1,2-C_2_B_9_H_11_)_2_]_y_ were established by ^1^H-NMR upon integration because the C_cluster_-H proton atoms of [3,3′-Co(1,2-C_2_B_9_H_11_)_2_]^−^ were easily identified (3.94 ppm in d_6_-acetone) and were weighted with regard to singular proton atoms, equally well identified, from the cation. The electroactive ion-pair complex made this way was so simple that practically no other spectroscopic or elemental analysis technique was required, but in many cases, Nuclear Magnetic Resonance (^1^H{^11^B}, ^11^B, ^11^B{1H} ^13^C{1H}) NMR, Fourier Transform Infrared Spectroscopy (FTIR), Elemental Analysis (EA), MALDI-TOF-MS spectroscopies were also used as further characterization (Table 1).

## 4. Results

As is common theory, the performance of ISEs is given by several parameters: the slope of the calibration curve, the linear working range, the limit of detection (LOD), selectivity and response time. These parameters strongly depend on the composition, stability, and reproducibility of the membrane and these derived from the [YH]_x_[3,3′-Co(1,2-C_2_B_9_H_11_)_2_]_y_ methodology are reported in Table 1.

The value of the slope of the calibration curve has to be in correlation with the number of charged species, proof of the Nernstian response of the prepared membranes. One of the most important components of the membrane composition is the nature (polar or nonpolar) and percentage of plasticizer, which ensure and improve the stability in time, the sensitivity, and selectivity.

pH is an important analytical parameter with a direct influence on sensitivity and selectivity. In our studies the influence of the pH on the ISEs answer in terms of Nernstian response and linear concentration range and for selectivity of the prepared membrane towards the analyte versus different interferences was done.

Commonly, the stability of the prepared membranes, in terms of slope was monitored for a period of 45 days. In this time, it was observed that the prepared membranes remain functional without degradation in performances for all of the studied analytes. The [*o*-COSAN]^−^ ISEs correlate very well with those traditionally made with ionophores taking advantage of its ease of preparation and very favorably of the lack of ionophore design and hence synthetic simplicity.

Another important parameter for ISEs is the response time, the time that the electrode needs until reaching a stable potential, which is directly correlated with the membrane thickness. For most of the membranes prepared following the protocol described in these papers the response time was around 5 s.

From the analytical point of view, selectivity is one of the most important parameters, especially when it is necessary to determine the target compounds from a complex matrix. In these studies, it was observed that a controlled tuning of the chemical composition of the membrane, in terms of ratio between the ion pair complex and plasticizers and the nature of plasticizers was possible to improve the selectivity

The potentiometric selectivity coefficients (Kpot_A/B_) were calculated based on the Nikolsky Eisenman equation [68,69] using the fixed interferences method (FIM). For each studied analyte, the selectivity of the prepared ISEs were tested for the possible inorganic and organic interference compounds and it was observed that the PVC membrane based on ion-pair complexes made between [*o*-COSAN]^−^ and the protonated tested analyte assured a good selectivity. The advantages of using [3,3′-Co(1,2-C_2_B_9_H_11_)_2_]^−^ as an ion-pair generator compared with other anions are based on: its unique 3D aromaticity [70], its high chemical and thermal stability (withstanding strong acid, moderate base, high temperatures and intense radiation) [71,72], as well as its biological stability (neither degradation nor chemical modification compounds were identified after cells’ uptake) [73,74], its lipophilicity [75], low-charge density [76], small volume molecules with a size of 1.1 × 0.6 nm [77], capacity to produce B–H···H–N dihydrogen bonds [19,77,78,79], and B–H···O, C_cluster_–H···N and B-H···Na or B-H···K hydrogen bonds [80,81,82] as well as unconventional cooperative effect such as C_cluster_−H···S−H···H−B hydrogen/dihydrogen bond interaction [83] that enhance the membrane stability over the time a fact that directly correlates with the sensitivity of the analyte determination. Further to these stability enhancing characteristics, it is our belief that the fact that the anion is redox reversible really influences in the good performances of these [3,3′-Co(1,2-C_2_B_9_H_11_)_2_]^−^ dependent electroactive ion-pair species.

## 5. Why Do These Membranes with Metallabis(dicarbollides), Being So Simple in Their Composition, Give Such Excellent Results?

Our explanation is simple; it is due to the θ-shape structure and chemical composition of the cobaltabis(dicarbollide) that give it unique properties. For instance, if we compare tetraphenylborate and [1-HCB_11_Br_11_]^−^ described in the paper as lipophilic ion-exchangers with [3,3′-Co(1,2-C_2_B_9_H_11_)_2_]^−^ it is noted that in the case of borate there are no B-H bonds but B-Br bonds, and the same for the tetraphenylborate that have C-H bonds so these cannot generate hydrogen and dihydrogen bonds. We believe this is essential. Thereby neither the one nor the other will generate strong interactions with the ammonium cation or with the plasticizer solvent. Therefore, they have high mobility in the membrane. This is not the case with [3,3′-Co(1,2-C_2_B_9_H_11_)_2_]^−^, which does make these strong interactions, and therefore in our view, generates more reticulate, and therefore more stability and a higher fixation of the concentration of the analyte in the membrane. The cobaltabis(dicarbollide) has dimensions of 1.1 nm in length and 0.6 nm in width and is surrounded by hydrogens, the mentioned B-H bonds, that have considerable hydride character but not enough to be unstable in protic solvents. This sufficient hydride character of the B-H groups enables it to interact strongly with H-N units. The non-bonding interactions are weak, but if there are many, they become a strong interaction. Surely, this is what makes it unnecessary to have ionophores in these membranes and that, on the other hand, the common ion to be detected that is present on both sides of the interface remains constant within the membrane giving the appropriate stability and sensitivity. In this case, we do not measure the anion, the cobaltabis(dicarbollide); we measure the cation.

## 6. Conclusions

Typically, ISEs for cations or anions require a ligand to complex them and thus achieve target selectivity. This ligand required a design and synthetic process that was usually laborious because it required the formation constant with the target ion to be much higher than an interfering one. This selectivity was also associated with a low dissociation constant, which could lead to difficulties in transferring information between the analyte phase and the internal phase. Our knowledge and understanding of the characteristics of [*o*-COSAN]^−^ and its high appetence for protonated amines as well as its solubility properties in aqueous media and in THF indicated that it could generate very efficient and easy to produce ionic pairs to detect amine cations, which made it very interesting for the determination of pharmaceuticals. The different potentials for the different redox couples in which [*o*-COSAN]^−^ could participate also made us believe that this system could facilitate information transfer between the analyte phase and the internal phase of the electrode. We could not observe the latter property as we did not exceed the 10^−6^ M detection limit, but we did find a very versatile membrane with a cationic electroactive substance, which was the one we wanted to detect. This cation was compensated by the anionic cobaltabis(dicarbollides), [cation-NH]_x_[3,3′-Co(1,2-C_2_B_9_H_11_)_2_]_y_, which allowed us to easily adapt it to the target we wanted to investigate. The strong interactions between the electroactive cation and [*o*-COSAN]^−^ and between [*o*-COSAN]^−^ and the plasticizer solvent favored a highly stable system very suitable for detecting amines as indicated in this work. The system is highly extrapolated to different amines, including enantiomers, which it detects in a clearly discriminatory way with respect to their optical isomers.

Thus, we developed membranes based on ion pair complexes between metallabis(dicarbollides), [3,3′-Co(1,2-C_2_B_9_H_11_)_2_]^−^, and bioactive protonable nitrogen containing compounds, [cation-NH]_x_[3,3′-Co(1,2-C_2_B_9_H_11_)_2_]_y_ that have proven that the properties of this anion open new directions for using these ISEs in environmental, clinical, pharmaceutical and food application and for miniaturization and mass production for routine analysis. 

## Figures and Tables

**Table 1:** Analytical performances of ISEs prepared for [cation-NH]_x_[3,3′-Co(1,2-C_2_B_9_H_11_)_2_]_y_. The plasticizer is abbreviated as: *o*-nitro phenyl octyl ether (NPOE); di-octyl phthalate (DOP); di-butyl phthalate (DBP); di-octyl sebacate (DOS), bis(2-ethylhexyl) phthalate (DEHP); tributyl phosphate (TBP).

Samples	Ion Pair Complex Formula	Plasticizer	Slope (mV·Decade^−1^)	Concentration Range (mol·dm^−3^)	Detection Limit (mol·dm^−3^)	Reference
Isoniazid	[H_3_INH][*o*-COSAN]_3_	NPOE	52.37	1.00 × 10^−4^– 1.00 × 10^−1^	5.00 × 10^−5^	[49]
Isoniazid	[H_3_INH][*o*-COSAN]_3_	DOP	47.80	1.00 × 10^−4^– 1.00 × 10^−1^	5.80 × 10^−5^	[49]
Pyrazinamide	H[HPZA]_2_[*o*-COSAN]_3_	NPOE	56.98	5.00 × 10^−4^– 1.00 × 10^−1^	3.00 × 10^−5^	[49]
Pyrazinamide	H[HPZA]_2_[*o*-COSAN]_3_	DOP	46.70	5.00 × 10^−5^– 1.00 × 10^−1^	1.00 × 10^−5^	[49]
L-Arginine	[HArg][*o*-COSAN]	DBP	45.80	5.00 × 10^−6^– 1.00 × 10^−1^	3.00 × 10^−6^	[50]
L-Arginine	[HArg][*o*-COSAN]	DEHP	37.70	1.00 × 10^−5^– 1.00 × 10^−1^	5.00 × 10^−5^	[50]
D-Histidine	[H_2_His][*o*-COSAN]_2_	DBP	36.50	1.00 × 10^−5^– 1.00 × 10^−1^	8.00 × 10^−6^	[50]
D-Histidine	[H_2_His][*o*-COSAN]_2_	DEHP	42.40	5.00 × 10^−5^– 1.00 × 10^−1^	2.00 × 10^−5^	[50]
L-Histidine	[H_2_His][*o*-COSAN]_2_	DBP	47.40	5.00 × 10^−6^– 1.00 × 10^−1^	1.00 × 10^−6^	[50]
L-Histidine	[H_2_His][*o*-COSAN]_2_	DEHP	48.50	5.00 × 10^−4^– 1.00 × 10^−1^	1.00 × 10^−4^	[50]
D-Tryptophan	[HTry][*o*-COSAN]	DBP	60.50	5.00 × 10^−7^– 1.00 × 10^−1^	2.00 × 10^−7^	[50]
L-Tryptophan	[HTry][*o*-COSAN]	DBP	62.60	5.00 × 10^−7^– 1.00 × 10^−1^	1.00 × 10^−7^	[50]
Dopamine	[HDA][*o*-COSAN]	Dibutylsebacate	44.97 ± 1.23	1.00 × 10^−6^– 1.00 × 10^−2^	0.80 × 10^−6^	[51]
Dopamine	[HDA][*o*-COSAN]	Bis (2-ethyl hexyl) phthalate	53.23 ± 1.75	1.00 × 10^−5^– 1.00 × 10^−2^	7.20 × 10^−6^	[51]
Dopamine	[HDA][*o*-COSAN]	NPOE	58.17 ± 1.44	5.00 × 10^−6^– 1.00 × 10^−2^	1.00 × 10^−6^	[51]
Dopamine	[HDA][*o*-COSAN]	DOP	55.96 ± 0.85	5.00 × 10^−6^– 1.00 × 10^−2^	1.00 × 10^−6^	[51]
Nicotinamide	[HNAmd][*o*-COSAN]	DOP	52.11 ± 1.17	5.00 × 10^−6^– 1.00 × 10^−2^	1.00 × 10^−6^	[51]
Nicotinic acid	[HNA][*o*-COSAN]	DOP	57.55 ± 0.88	1.00 × 10^−6^– 1.00 × 10^−2^	0.70 × 10^−6^	[51]
Histamine	[H_2_His][*o*-COSAN]_2_	NPOE	31.62 ± 0.43	5.00 × 10^−6^– 1.00 × 10^−2^	0.80 × 10^−6^	[51]
Metformin	[H_2_Met][*o*-COSAN]_2_	NPOE	25.82 ± 1.91	1.00 × 10^−5^– 1.00 × 10^−2^	6.00 × 10^−6^	[51]
Serotonin	[HSer][*o*-COSAN]	DBS	50.50 ± 0.50	2.25 × 10^−5^– 1.00 × 10^−2^	4.51 × 10^−6^	[52]
Serotonin	[HSer][*o*-COSAN]	DBP	50.60 ± 0.50	2.25 × 10^−5^– 1.00 × 10^−2^	1.17 × 10^−5^	[52]
Serotonin	[HSer][*o*-COSAN]	TBP	60.50 ± 0.30	2.25 × 10^−5^– 1.00 × 10^−2^	1.56 × 10^−5^	[52]
Serotonin	[HSer][*o*-COSAN]	NPOE	51.10 ± 0.10	2.25 × 10^−5^– 1.00 × 10^−2^	1.70 × 10^−5^	[52]
Irgarol	[Irg-H][*o*-COSAN]	DOP	56.67 ± 2.30	1.00 × 10^−5^– 1.00 × 10^−1^	3.00 × 10^−6^	[53]
Irgarol	[Irg-H][*o*-COSAN]	DOS	57.17 ± 1.70	1.00 × 10^−5^– 1.00 × 10^−1^	2.00 × 10^−6^	[53]
Irgarol	[Irg-H][*o*-COSAN]	NPOE	48.21 ± 6.40	1.00 × 10^−5^– 1.00 × 10^−1^	4.00 × 10^−6^	[53]
Sulfapyridine	A-H][*o*-COSAN]	NPOE	47.69	1.00 × 10^−6^– 1.00 × 10^−3^	4.00 × 10^−6^	[54]
Sulfapyridine	[A-H][*o*-COSAN]	DOS	61.29	1.00 × 10^−6^– 1.00 × 10^−3^	1.00 × 10^−6^	[54]
Sulfapyridine	[A-H][*o*-COSAN]	DOP	61.26	1.00 × 10^−6^– 1.00 × 10^−3^	1.00 × 10^−5^	[54]
Amphetamine	[Amph-H][*o*-COSAN]	DBP	60	1.00 × 10^−5^– 1.00 × 10^−3^	12.00 × 10^−6^	[55]
Amphetamine	[Amph-H][*o*-COSAN]	DOP	42	1.00 × 10^−5^– 1.00 × 10^−3^	8.00 × 10^−6^	[55]
Amphetamine	[Amph-H][*o*-COSAN]	DOS	53	1.00 × 10^−5^– 1.00 × 10^−3^	4.00 × 10^−5^	[55]
Amphetamine	[Amph-H][*o*-COSAN]	NPOE	45	1.00 × 10^−5^– 1.00 × 10^−3^	2.00 × 10^−5^	[55]
Antipyrine	[AP][*o*-COSAN]	NPOE	79.6 ± 4.9	1.00 × 10^−5^– 1.00 × 10^−2^	70.8 × 10^−6^ ± 9.3	[56]
Antipyrine	[AP][*o*-COSAN]	DBS	57.0 ± 1.4	1.00 × 10^−5^– 1.00 × 10^−2^	29.8 × 10^−6^ ± 2.2	[56]
4-(methylamino) antipyrine	[MAAP][*o*-COSAN]	NPOE	33.9 ± 1.0	1.00 × 10^−5^– 1.00 × 10^−2^	27.3 × 10^−6^ ± 1.5	[56]
4-(methylamino)antipyrine	[MAAP][*o*-COSAN]	DBS	48.2 ± 1.0	1.00 × 10^−5^– 1.00 × 10^−2^	279.5 × 10^−6^ ± 7.0	[56]
4-aminoantipyrine	[AAP][*o*-COSAN]	NPOE	54.6 ± 3.8	1.00 × 10^−5^– 1.00 × 10^−2^	88.2 × 10^−6^ ± 14.8	[56]
4-aminoantipyrine	[AAP][*o*-COSAN]	DBS	71.2 ± 6.1	1.00 × 10^−5^– 1.00 × 10^−2^	342.0 × 10^−6^ ± 27.2	[56]
4-acetamidoantipyrine	[AAAP][*o*-COSAN]	NPOE	57.0 ± 2.0	1.00 × 10^−5^– 1.00 × 10^−2^	252.2 × 10^−6^ ± 18.7	[56]

## Data Availability

Not applicable.

## References

[1] Kerru N., Gummidi L., Maddila S., Gangu K.K., Jonnalagadda S.B. (2020). A Review on Recent Advances in Nitrogen-Containing Molecules and Their Biological Applications. Molecules.

[2] Heravi M.M., Zadsirjana V. (2020). Prescribed Drugs Containing Nitrogen Heterocycles: An Overview. RSC Adv..

[3] Siddiqui M.R., AlOthman Z.A., Rahman N. (2017). Analytical techniques in pharmaceutical analysis: A review. Arab. J. Chem..

[4] Xu Q., Yuan A.J., Zhang R., Bian X., Chen D., Hu X. (2009). Application of Electrochemical Methods for Pharmaceutical and Drug Analysis. Curr. Pharm. Anal..

[5] Mostafa I.M., Meng C., Dong Z., Lou B., Xu G. (2022). Potentiometric Sensors for the Determination of Pharmaceutical Drugs. Anal. Sci..

[6] Kharitonov S.V. (2007). Ion-Selective Electrodes in Medicinal Drug Determination. Russ. Chem. Rev..

[7] Tong H.Y., Meng J., Liang J.Y., Li J.P. (2021). Molecularly Imprinted Electrochemical Luminescence Sensor Based on Core-Shell Magnetic Particles with ZIF-8 Imprinted Material. Sens. Actuators B Chem..

[8] Li X.H., Kuang X.J., Sun J.L. (2021). Rare Earth Elements Based Oxide Ion Conductors. Inorg. Chem. Front..

[9] Yao M.M., Huang J.K., Deng Z.H., Jin W.Y., Yuan Y.L., Nie J.F., Wang H., Du F.Y., Zhang Y. (2020). Transforming Glucose into Fluorescent Graphene Quantum Dots Via Microwave Radiation for Sensitive Detection of Al^3+^ Ions Based on Aggregation-Induced Enhanced Emission. Analyst.

[10] Hawthorne M.F., Andrews T.D. (1965). Carborane Analogues of Cobalticinium Ion. Chem. Commun..

[11] Lever A.B.P. (1990). Electrochemical Parametrization of Metal Complex Redox Potentials, using the Ruthenium(III)/Ruthenium(II) Couple to Generate a Ligand Electrochemical Series. Inorg. Chem..

[12] Morris J.H., Gysling H.J., Reed D. (1985). Electrochemistry of Boron Compounds. Chem. Rev..

[13] Núñez R., Tarrés M., Ferrer-Ugalde F., de Biani F.F., Teixidor F. (2016). Electrochemistry and Photoluminescence of Icosahedral Carboranes, Boranes, Metallacarboranes, and their Derivatives. Chem. Rev..

[14] Bauduin P., Prevost S., Farras P., Teixidor F., Diat O., Zemb T. (2011). A Theta-Shaped Amphiphilic Cobaltabisdicarbollide Anion: Transition from Monolayer Vesicles to Micelles. Angew. Chem. Int. Ed..

[15] Malaspina D.C., Viñas C., Teixidor F., Faraudo J. (2020). Atomistic Simulations of COSAN: Amphiphiles without a Head-and-Tail Design Display “Head and Tail” Surfactant Behavior. Angew. Chem. Int. Ed..

[16] Juárez-Pérez E.J., Núñez R., Viñas C., Sillanpää R., Teixidor F. (2010). The Role of C–H···H–B Interactions in Establishing Rotamer Configurations in Metallabis (dicarbollide) Systems. Eur. J. Inorg. Chem..

[17] Farràs P., Juárez-Pérez E.J., Lepšík M., Luque R., Núñez R., Teixidor F. (2012). Metallacarboranes and their Interactions: Theoretical Insights and their Applicability. Chem. Soc. Rev..

[18] Verdiá-Báguena C., Alcaraz A., Aguilella V.M., Cioran A.M., Tachikawa S., Nakamura H., Teixidor F., Viñas C. (2014). Amphiphilic COSAN and I2-COSAN Crossing Synthetic Lipid Membranes: Planar Bilayers and Liposomes. Chem. Commun..

[19] Fuentes I., Pujols J., Viñas C., Ventura S., Teixidor F. (2019). Dual Binding Mode of Metallacarborane Produces a Robust Shield on Proteins. Chem. Eur. J..

[20] de Marco R., Clarke G., Pejcic B. (2007). Ion-Selective Electrode Potentiometry in Environmental Analysis. Electroanalysis.

[21] Mikhelson K.N. (2013). Ion-Selective Electrodes.

[22] Bakker E., Pretsch E. (2007). The New Wave of Ion-Selective Electrodes. Anal. Chem..

[23] Bakker E., Pretsch E. (2007). Modern Potentiometry. Angew. Chem. Int. Ed..

[24] Lewenstam A. (2014). Routines and Challenges in Clinical Application of Electrochemical Ion-Sensors. Electroanalysis.

[25] Bloch R., Shatkay A., Saroff H.A. (1967). Fabrication and Evaluation of Membranes as Specific Electrodes for Calcium Ions. Biophys. J..

[26] Moody G.J., Oke R.B., Thomas J.D.R. (1970). A Calcium-Sensitive Electrode Based on a Liquid Ion Exchanger in a Poly (Vinyl Chloride) Matrix. Analyst.

[27] Stefanac Z., Simon W. (1967). Ion Specific Electrochemical Behavior of Macrotetrolides in Membranes. Microchem. J..

[28] Viñas C., Gómez S., Bertran J., Barron J., Teixidor F., Dozol J.-F., Rouquette H., Kivekkäs R., Sillanpää R. (1999). C-substituted bis (dicarbollide) metal compounds as sensors and extractants of radionuclides from nuclear wastes. J. Organomet. Chem..

[29] Kopytin A.V., Zhizhin K.Y., Urusov Y.I., Mustyatsa V.N., Kokunov Y.V., Kuznetsov N.T. (2012). Potentiometric Sensors with Membranes Based on Ionic Liquid Tetradecylammonium Triethylammonio-closo-Dodecaborate. J. Anal. Chem..

[30] Kubasov A.S., Turishev E.S., Kopytin A.V., Shpigun L.K., Zhizhin K.Y., Kuznetsov N.T. (2021). Sulfonium closo-hydridodecaborate Anions as Active Components of a Potentiometric Membrane Sensor for Lidocaine Hydrochloride. Inorg. Chim. Acta..

[31] Peper S., Telting-Diaz M., Almond P., Albrecht-Schmitt T., Bakker A. (2002). Perbrominated closo-Dodecacarborane Anion, [1-HCB_11_Br_11_]^−^, as an Ion Exchanger in Cation-Selective Chemical Sensors. Anal. Chem..

[32] Bakker E., Nägele M., Schaller U., Pretsch E. (1995). Applicability of the Phase Boundary Potential Model to the Mechanistic Understanding of Solvent Polymeric Membrane-Based Ion-Selective Electrodes. Electroanalysis.

[33] Karpfen F.M., Randles J.E.B. (1953). Ionic Equilibria and Phase-Boundary Potentials in Oil-Water Systems. Trans. Faraday Soc..

[34] Núñez R., Romero I., Teixidor F., Viñas C. (2016). Icosahedral Boron Clusters: A Perfect Tool for the Enhancement of Polymer Features. Chem. Soc. Rev..

[35] Peper S., Qin Y., Almond P., McKee M., Telting-Diaz M., Albrecht-Schmitt T., Bakker E. (2003). Ion-Pairing Ability, Chemical Stability, and Selectivity Behavior of Halogenated Dodecacarborane Cation Exchangers in Neutral Carrier-Based Ion-Selective Electrodes. Anal. Chem..

[36] Bakker E., Pretsch E. (1995). Lipophilicity of Tetraphenylborate Derivatives as Anionic Sites in Neutral Carrier-Based Solvent Polymeric Membranes and Lifetime of Corresponding Ion-Selective Electrochemical and Optical Sensors. Anal. Chim. Acta.

[37] Ortuno J.A., Rodenas V., Garcıa M.S., Albero M.I., Sanchez-Pedreno C.A. (2007). New Tiapride Selective Electrode and Its Clinical Application. Sensors.

[38] Malongo K., Blankert B., Kambu O., Amighi K., Nsangu J., Kauffmann J.M. (2006). Amodiaquine Polymeric Membrane Electrode. J. Pharm. Biomed. Anal..

[39] Aboul-Enein H.Y., Sun X.X., Sun C.J. (2002). Ion Selective PVC Membrane Electrode for the Determination of Methacycline Hydrochloride in Pharmaceutical Formulation. Sensors.

[40] Kulapina E.G., Barinova O.V. (2001). Ion-Selective Electrodes for the Determination of Nitrogen-Containing Medicinal Substances. J. Anal. Chem..

[41] Ortuño J.A., Hernández J., Sánchez-Pedreño C. (2006). Ion-Selective Electrode for the Determination of Some Multidrug Resistance Reversers. Sens. Actuators B Chem..

[42] Bakker E., Pretsch E., Bühlmann P. (2000). Selectivity of Potentiometric Ion Sensors. Anal. Chem..

[43] Freiser H. (2012). Ion-Selective Electrodes in Analytical Chemistry.

[44] Bakker E., Chumbimuni-Torres K. (2008). Modern Directions for Potentiometric Sensor. J. Braz. Chem. Soc..

[45] Guggenheim E.A. (1929). The Conceptions of Electrical Potential Difference between Two Phases and the Individual Activities of Ions. J. Phys. Chem..

[46] Guggenheim E.A. (1930). On the Conception of Electrical Potential Difference between two Phases. II. J. Phys. Chem..

[47] Theorell T. (1935). An Attempt to Formulate a Quantitative Theory of Membrane Permeability. Proc. Soc. Exp. Biol. Med..

[48] Meyer K.H., Sievers J.F. (1936). La Perméabilité des Membranes I. Théorie de la Perméabilité Ionique. Helv. Chim. Acta.

[49] Stoica A.I., Vinas C., Teixidor F. (2008). Application of the Cobaltabisdicarbollide Anion to the Development of Ion Selective PVC Membrane Electrodes for Tuberculosis Drug Analysis. Chem. Commun..

[50] Stoica A.I., Vinas C., Teixidor F. (2009). Cobaltabisdicarbollide Anion Receptor for Enantiomer-Selective Membrane Electrodes. Chem. Commun..

[51] Stoica A.I., Kleber C., Vinas C., Teixidor F. (2013). Ion Selective Electrodes for Protonable Nitrogen Containing Analytes: Metallacarboranes as Active Membrane Components. Electrochim. Acta.

[52] Bliem C., Fruhmann P., Stoica A.I., Kleber C. (2017). Development and Optimization of an Ion-selective Electrode for Serotonin Detection. Electroanalysis.

[53] Saini A., Gallardo-Gonzalez J., Baraket A., Fuentes I., Viñas C., Zine N., Bausells J., Teixidor F., Errachid A. (2018). A Novel Potentiometric Microsensor for Real-Time Detection of Irgarol using the Ion-Pair Complex [Irgarol-H]^+^[Co(C_2_B_9_H_11_)_2_]^−^. Sens. Actuators B Chem..

[54] Saini A., Fuentes I., Viñas C., Zine N., Bausells J., Errachid A., Teixidor F. (2019). A Simple Membrane with the Electroactive [Sulfapyridine-H]^+^[Co(C_2_B_9_H_11_)_2_]^−^ for the Easy Potentiometric Detection of Sulfonamides. J. Organomet. Chem..

[55] Gallardo-Gonzalez J., Saini A., Baraket A., Boudjaoui S., Alcácer A., Strelas A., Teixidor F., Zine N., Bausells J., Errachid A. (2018). A Highly Selective Potentiometric Amphetamine Microsensor Based on All-Solid-State Membrane Using a New Ion-Pair Complex, [3,3′-Co(1,2-closo-C_2_B_9_H_11_)_2_]^−^[C_9_H_13_NH]^+^. Sens. Actuators B Chem..

[56] Mayerhuber L., Trattner S., Luger S., Weigelhofer G., Hametner C., Fruhmann P. (2021). Development of Ion-Selective Electrodes for Antipyrine and its Derivatives as Potential Tool for Environmental Water Monitoring. J. Electroanal. Chem..

[57] Thoma A.P., Cimerman Z., Fiedler U., Bedekovic D., Güggi M., Jordan P., May K., Pretsch E., Prelog V., Simon W. (1975). Enantiomerenselektives Verhalten in Membranen Eines Chiralen Elektrisch Neutralen Ionophores. Chimia.

[58] Trojanowicz M. (2014). Enantioselective Electrochemical Sensors and Biosensors: A Mini-Review. Electrochem. Commun..

[59] Trojanowicz M., Kaniewska M. (2009). Electrochemical Chiral Sensors and Biosensors. Electroanalysis.

[60] Yasaka Y., Yamamoto T., Kimura K., Shono T. (1980). Simple Evaluation of Enantiomer-Selectivity of Crown Ether Using Membrane Electrode. Chem. Lett..

[61] Maruyama K., Sohmiya H., Tsukube H. (1989). New Chiral Host Molecules Derived from Naturally Occurring Monensin Ionophore. J. Chem. Soc. Chem. Commun..

[62] Maruyama K., Sohmiya H., Tsukube H. (1992). Enantiomer Recognition of Organic Ammonium Salts by Podand and Crown-e Monensin Amides: New Synthetic Strategy for Chiral Receptors. Tetrahedron.

[63] Kaniewska M., Sikora T., Kataky R., Trojanowicz M. (2008). Enantioselectivity of Potentiometric Sensors with Application of Different Mechanisms of Chiral Discrimination. J. Biochem. Biophys. Methods.

[64] Ji J., Qu L., Wang Z., Li G., Feng W., Yang G. (2022). A facile electrochemical chiral sensor for tryptophan enantiomers based on multiwalled carbon nanotube/hydroxypropyl-β-cyclodextrin functionalized carboxymethyl cellulose. Microchem. J..

[65] Liua N., Yang B., Yin Z.-Z., Cai W., Li J., Kong Y. (2022). A chiral sensing platform based on chiral metal-organic framework for enantiodiscrimination of the isomers of tyrosine and tryptophan. J. Electroanal. Chem..

[66] Lu Q., Chen L., Meng Q., Jiang Y., Xie L. (2021). A biomolecule chiral interface base on BSA for electrochemical recognition of amine enantiomers. Chirality.

[67] Luger S., Mayerhuber L., Weigelhofer G., Hein T., Holzer B., Hametner C., Fruhmann P. (2022). Development of Ion-selective Electrodes for Tropane, Atropine, and Scopolamine—A Concept for the Analysis of Tropane Alkaloids. Electroanalysis.

[68] Analytical Chemistry Division, Commission on Analytical Nomenclature (1976). Recommendations for Nomenclature of Ion-Selective Electrodes, “Recommendations–1975”. Pure Appl. Chem..

[69] Buck R.P., Lindner E. (1994). Recommendations for Nomenclature of Ion-Selective Electrodes IUPAC Recommendations 1994. Pure Appl. Chem..

[70] Poater J., Viñas C., Bennour I., Gordils S.E., Sola M., Teixidor F. (2020). Too Persistent to Give Up: Aromaticity in Boron Clusters Survives Radical Structural Changes. J. Am. Chem. Soc..

[71] Grimes R.N. (2016). Carboranes.

[72] Plesek J. (1992). Potential applications of the boron cluster compounds. Chem. Rev..

[73] Tarrés M., Canetta E., Paul E., Forbes J., Azzouni K., Viñas C., Teixidor F., Harwood A.J. (2015). Biological interaction of living cells with COSAN-based synthetic vesicles. Sci. Rep..

[74] Fuentes I., García-Mendiola T., Sato S., Pita M., Nakamura H., Lorenzo E., Teixidor F., Marques F., Viñas C. (2018). Metallacarboranes on the Road to Anticancer Therapies: Cellular Uptake, DNA Interaction, and Biological Evaluation of Cobaltabisdicarbollide [COSAN]−. Chem. Eur. J..

[75] Bennour I., Ramos M.N., Nuez-Martínez M., Xavier J.A.M., Buades A.B., Sillanpää R., Teixidor F., Choquesillo-Lazarte D., Romero I., Martinez-Medina M. (2022). Water soluble organometallic small molecules as promising antibacterial agents: Synthesis, physical–chemical properties and biological evaluation to tackle bacterial infections. Dalton Trans..

[76] Masalles C., Llop J., Vinas C., Teixidor F. (2002). Extraordinary overoxidation resistance increase in self-doped polypyrroles by using non-conventional low charge-density anions. Adv. Mater..

[77] Goszczynski T.M., Fink K., Kowalski K., Lesnikowski Z.J., Boratynski J. (2017). Interactions of Boron Clusters and their Derivatives with Serum Albumin. Sci. Rep..

[78] Cıgler P., Kozısek M., Rezacova P., Brynda J., Otwinowski Z., Pokorna J., Plesek J., Grüner B., Doleckova-Maresova L., Masa M. (2005). From nonpeptide toward noncarbon protease inhibitors: Metallacarboranes as specific and potent inhibitors of HIV protease. Proc. Natl. Acad. Sci. USA.

[79] Fanfrlık J., Brynda J., Rezac J., Hobza P., Lepsık M. (2008). Interpretation of Protein/Ligand Crystal Structure using QM/MM Calculations: Case of HIV-1 Protease/Metallacarborane Complex. J. Phys. Chem. B.

[80] Matějíček P., Zedník J., Ušelová K., Pleštil J., Fanfrlík J., Nykänen A., Ruokolainen J., Hobza P., Procházka K. (2009). Stimuli-Responsive Nanoparticles Based on Interaction of Metallacarborane with Poly (ethylene oxide). Macromolecules.

[81] Tarrés M., Viñas C., Gonzalez-Cardoso P., Hänninen M.M., Sillanpää R., Dordovic V., Uchman M., Teixidor F., Matejicek P. (2014). Aqueous Self-Assembly and Cation Selectivity of Cobaltabisdicarbollide Dianionic Dumbbells. Chem. Eur. J..

[82] Teixidor F., Pedrajas J., Rojo I., Viñas C., Kivekäs R., Sillanpää R., Sivaev I., Bregadze V., Sjöberg S. (2003). Chameleonic Capacity of [3,3′-Co(1,2-C2B9H11)2]-in Coordination. Generation of the Highly Uncommon S (thioether)-Na Bond. Organometallics.

[83] Planas J.G., Viñas C., Teixidor F., Comas-Vives A., Ujaque G., Lledós A., Light M.E., Hursthouse M.B. (2005). Self-Assembly of Mercaptane−Metallacarborane Complexes by an Unconventional Cooperative Effect: A C−H···S−H···H−B Hydrogen/Dihydrogen Bond Interaction. J. Am. Chem. Soc..

